# Knowledge of first-aid management of traumatic dental injuries among secondary school students in Central Poland: a cross-sectional study

**DOI:** 10.1007/s44445-026-00126-2

**Published:** 2026-02-11

**Authors:** Karolina Spodzieja, Wiktoria Mól, Paula Piekoszewska–Ziętek, Marcin Studnicki, Dorota Olczak-Kowalczyk

**Affiliations:** 1 Department of Paediatric Dentistry, Medical University of Warsaw, Binieckiego 6 St., 02-097 Warsaw, Poland; 2 Department of Experimental Design and Bioinformatics, Warsaw University of Life Sciences, Nowoursynowska 159, 02-776 Warsaw, Poland

**Keywords:** Traumatic dental injuries, Dental trauma management, First aid education, Oral health awareness

## Abstract

Traumatic dental injuries (TDI) are a common problem especially in the field of paediatric dentistry and constitute a serious health issue in the young population. Prognosis of TDI treatment depend on various factors, for a long – term success immediate and proper first aid is crucial to preserve the vitality and function of damaged teeth. Since most cases of dental trauma occur at school – teachers and students are the first ones to encounter and to provide first aid in the emergency situation. Little attention is paid to educating primary and secondary school children about the first aid when the dental trauma occurs. The aim of our study was to investigate the knowledge of Polish secondary school students in management of traumatic injuries. A 34-item questionnaire on TDI experience and management was distributed amongst secondary school students attending different types of school in Poland. Statistical analyses were carried out using the IBM SPSS Statistics 25 package. It was used to perform Mann–Whitney’s U tests, Kruskal–Wallis non-parametric ANOVA and Spearman’s rank correlations. Two hundred and fifty eight questionnaires were obtained in the study. 76,7% of all responders have witnessed dental trauma in the past. 64,3% of them had suffered from TDI. Over 50% of students were unaware of a suitable medium in which to transport an avulsed permanent tooth. Only 32,2% decided that an avulsed tooth can be put back in its place. This study indicates a lack of adequate knowledge of management of TDI among secondary school students in Poland. Additional education about first aid of TDI should be developed and implemented. Appropriate first aid, emergency care at the accident site can save the patient more costly and time consuming treatment as well as negative health consequences including tooth loss.

## Introduction

Traumatic dental injuries (TDIs) are frequent in paediatric dentistry and represent an important health concern in children and adolescents. Compared with adults, young people experience dental trauma more often due to immature motor coordination, high levels of physical activity, participation in contact sports, and everyday accidents in school settings where they spend much of their time. Consequently, educational institutions are considered high-risk environments for TDIs (Skaare and Jacobsen 2003; Andersson 2013).

The clinical spectrum of dental trauma ranges from mild injuries—such as enamel cracks, concussion, or uncomplicated crown fractures—to severe damage involving periodontal tissues and alveolar bone, which may result in tooth displacement or avulsion. The International Association of Dental Traumatology (IADT) classifies these injuries and provides evidence-based recommendations for their management (Levin et al. 2020, 2024). Among children and adolescents, maxillary incisors are most commonly affected (Srilatha et al. 2021).

Treatment outcomes depend on multiple factors, including the type and severity of trauma, root development stage, the clinician’s expertise, the time elapsed since injury, the quality of on-site emergency measures, and appropriate follow-up care. Prompt, correct first aid is essential for preserving tooth vitality and function and improving long-term prognosis (Zaleckienė et al. 2018). Inadequate or delayed management may lead to complications such as pulpal inflammation or necrosis, malocclusion, periapical infection, ankylosis, root resorption, and ultimately tooth loss (Andreasen and Kahler 2015; Pohl et al. 2005). These sequelae—whether in the primary or permanent dentition—can negatively influence mastication, speech, aesthetics, psychosocial wellbeing, and overall quality of life.

Basic knowledge and simple protocols—such as haemostasis, immobilization, replantation of avulsed teeth, appropriate transport media for avulsed teeth, and preservation of tooth fragments—can prevent patients from requiring complex and costly dental procedures. (Tewari et al. 2021).

Because many dental injuries occur at school, teachers and students are often the first responders. Most published studies evaluate knowledge and attitudes toward TDI management among teachers, sports coaches, dental students, parents, or general practitioners. In contrast, far less attention has been given to educating schoolchildren themselves, despite their potential role in initiating immediate help. To our knowledge, studies assessing first-aid knowledge for dental trauma among adolescent students are lacking. Therefore, the aim of this study was to address this gap by investigating the knowledge of Polish secondary school students regarding first-aid management of traumatic dental injuries.

## Materials and methods

**Fig. 1:**
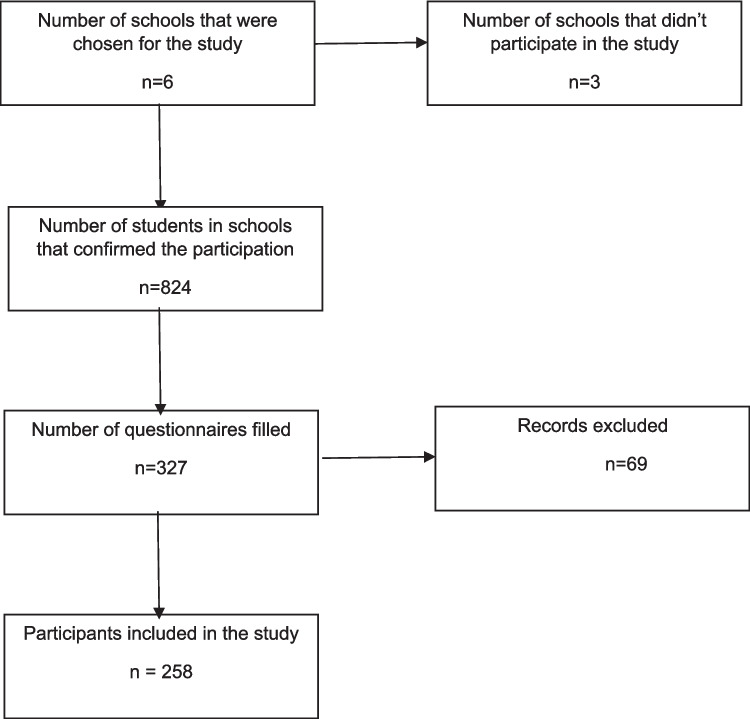
Methodology of the study

The study protocol was approved by the Bioethics Committee of Medical University of Warsaw (ref. no. AKBE/122/2022). The cross-sectional survey was conducted among 258 students aged 14–19 years attending three public secondary schools (general secondary, technical and vocational) located in central Poland. A convenience sample was used, and the study was not designed to be nationally representative. The minimum required sample size (n = 195) was calculated from the number of all secondary school students in Poland, with 95% confidence interval, 7% margin of error, and a population proportion of 50%. Contact was made with principals and teachers of secondary schools by email and telephone to present the research and obtain the consent form. A cover letter which explained the purpose of the study was sent to school principals with a link to an electronic survey. Six schools from different parts of central Poland were chosen to participate in the study. Methodology of the study is presented in Fig. 1. Those who agreed to participate in the study, answered an online questionnaire through the Google Forms platform. Questionnaire did not require names or contact information and the participants were ensured about the confidentiality of their information.

After thorough review of recent cross – sectional studies evaluating teacher’s and students’ knowledge in management of TDI a questionnaire consisting of 34 questions was designed. Three dental specialists evaluated the final version of questionnaire before sending it to secondary school. The survey was divided into three parts. Part I (Q1-Q6) included personal information and demographic characteristics of students and their parents, part II (Q7 – Q20) experience and management of traumatic injuries in the past, part III (Q21- Q34) knowledge of dental trauma management in general.

### Statistical analysis

In order to verify the research questions and research hypotheses, statistical analyses were carried out using the IBM SPSS Statistics 25 package. It was used to perform Mann–Whitney’s U tests, Kruskal–Wallis non-parametric ANOVA and Spearman’s rank correlations. The classic threshold of α = 0.05 was considered the level of significance.

## Results

A total of 258 secondary school students in Poland participated in this study. 65,5% (169) of them were male, 34,5% (89) were female. 73.6% (190) of all responders lived in the urban area, 26,4% (68) lived in the rural area. 49,2% (127) of them attended general high school, 34,9% (90) of them attended technical school and 15,9% (41) vocational school. Demographics of the responders are presented in Table 1.

**Table 1:** Demographic data of the study participants

		*N*	%
School	High school	127	49.2
	Technical school	90	34.9
	Vocational school	41	15.9
History of dental trauma	Absent	92	35.7
	Present	166	64.3
Mother education level	Primary	14	5.4
	Secondary	91	35.3
	Higher	153	59.3
Father education level	Primary	20	7.8
	Secondary	101	39.1
	Higher	137	53.1

79,8% of all surveyed students responded that in the past attended the first aid course, but only 7,1% of them stated that during the course TDI management and treatment was discussed.

76,7% of all responders have witnessed dental trauma in the past. 64,3% of them had suffered from TDI and 62,6% of them have received immediate dental treatment within a few hours. 18,4% of all responders who suffered from TDI in the past stated that dental injury occurred more than once. 45% of students suffered from dental injury at school or at the school playground, 48,4% at home and 6,6% in other circumstances. 63,9% of all responders practice sport in their leisure time, but only 11,5% of them use mouthguards.

Upper and lower permanent incisors have been mentioned to be most often affected in the injury. Many of them also stated that had injury to deciduous dentition in the past. 28,9% (48) of students who suffered from dental trauma had crown fracture, 22,9% (38) concussion or subluxation, 4,2% (7) extrusion, 3,6% (6) intrusion, 6,6% (11) root fracture, 6,6% (11) avulsion and 27,1% (45) of them could not specify the type of injury.

55% of respondents didn’t know that a fragment of a broken tooth can be useful in treatment and should be taken to the dentist. 47,7% thought that an extruded tooth should be removed. 32,2% decided that an avulsed tooth can be put back in its place. 73,9% of the responders answered that an avulsed tooth should be cleaned before reposition, 70,2% stated that it should be kept in a special medium. Over 50% of students were unaware of a suitable medium in which to transport an avulsed permanent tooth. Only 10,4% of them thought an avulsed tooth should be kept in saliva, 15% in milk and 33,3% of them decided it should be transported in a saline solution. 70% thought that people with malocclusion are more susceptible to dental injuries.

### Knowledge level of students

In the next step, we analyzed the level of knowledge. In Table 2 we presented percentage of correct answers for all seven knowledge test questions.

**Table 2:** Number and percentage of correct answers

	*N*	%
1. Are necessary regular dental check-ups after suffering a dental injury?	199	77.1
2. What should be done if a tooth after an injury is painful while pressing or eating, but is not mobile?	103	39.9
3. What should be done if the tooth is painful and mobile after an injury?	183	70.9
4. Can a fragment of a broken tooth still be useful in treatment and should be taken to the dentist?	116	45
5. Do you think that a tooth that is mobile after an injury and has slipped out of the socket (it is longer than the others) can be put back in place or does it have to be removed?	135	52.3
6. Do you think a completely dislocated tooth (i.e. the crown and root outside the mouth) can be put back in its place?	83	32.2
7. Do you think that an avulsed tooth should be kept in a special medium before you go to the dentist?	181	70.2

**Fig. 2:**
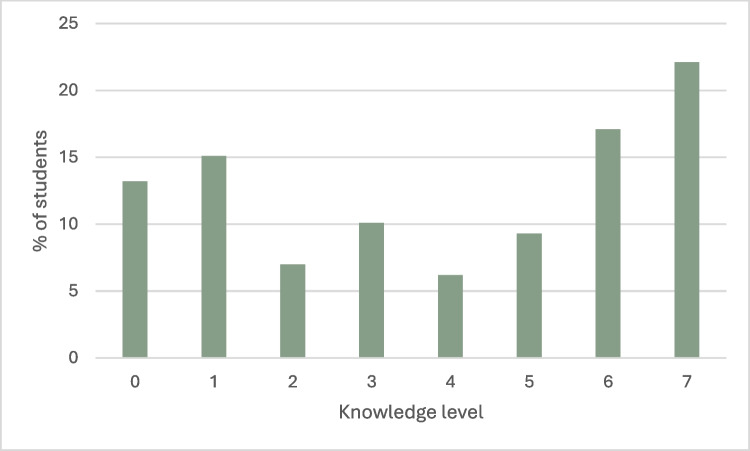
Knowledge level distribution among secondary school students

We calculated the knowledge level by summing the number of correct answers. Thus, the results ranged from 0 to 7 points. Mean value in analyzed group was 3.88 (*SD* = 2.57) while median value was 4 points. Distribution of results in all student’s group was presented on Fig. 2. The Shapiro–Wilk test was also performed to check the normality of the tested distribution. The distribution was found to be statistically significantly different from the normal distribution (*D* = 0.87; *p* < 0.001). Therefore, all analysis were calculated by means of non-parametric tests.

**Fig. 3:**
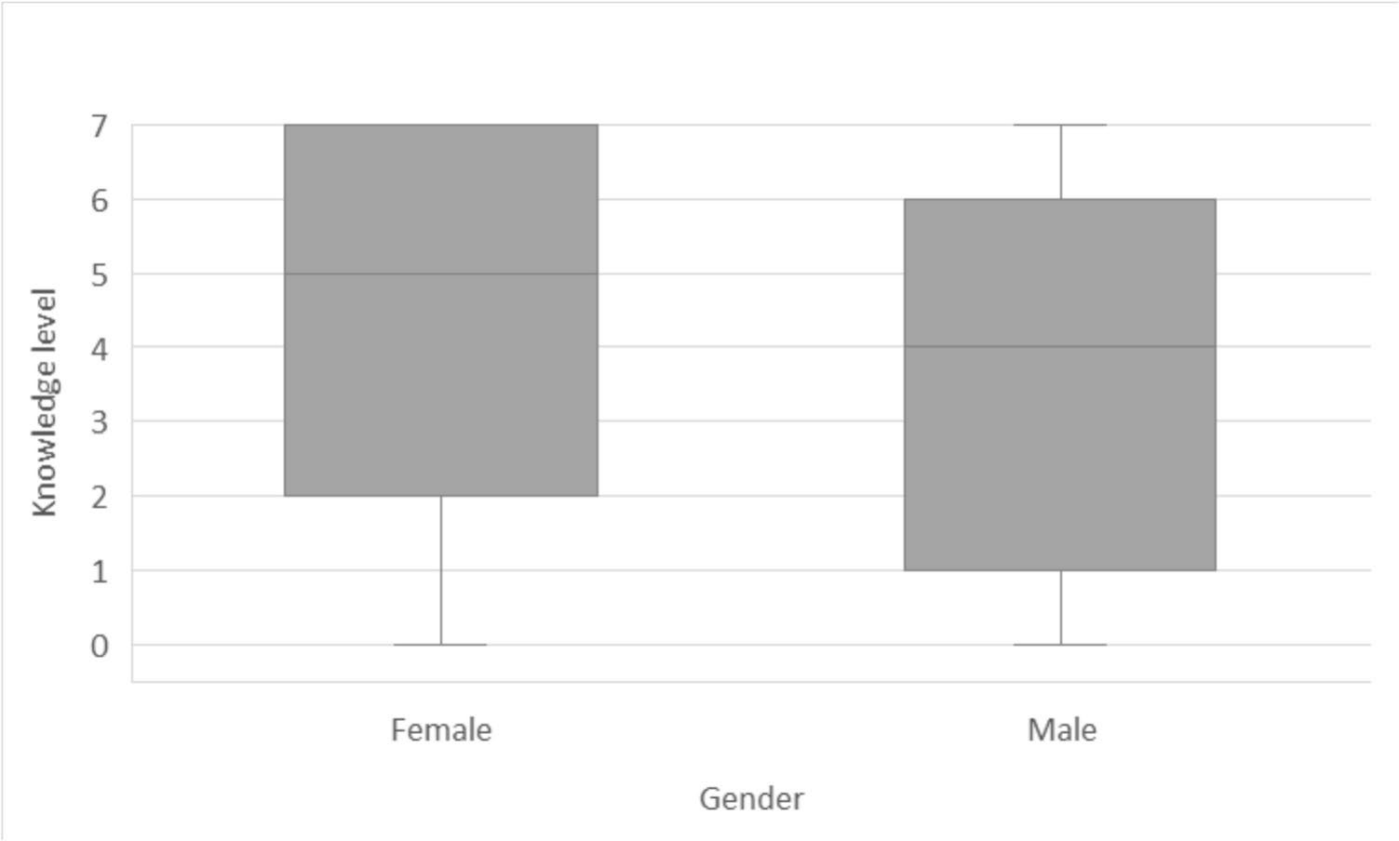
Knowledge level in female and male secondary school students

To verify the difference in knowledge level between female and male students, we performed *U* Mann–Whitney’s test, that happened to be statistically significant, *U* = 5534.5; *Z* = −3.53; *p* < 0.001; *r* = 0.22. Female students got higher results than male students. Size of observed effect was low. Results are presented in Fig. 3.

**Fig. 4:**
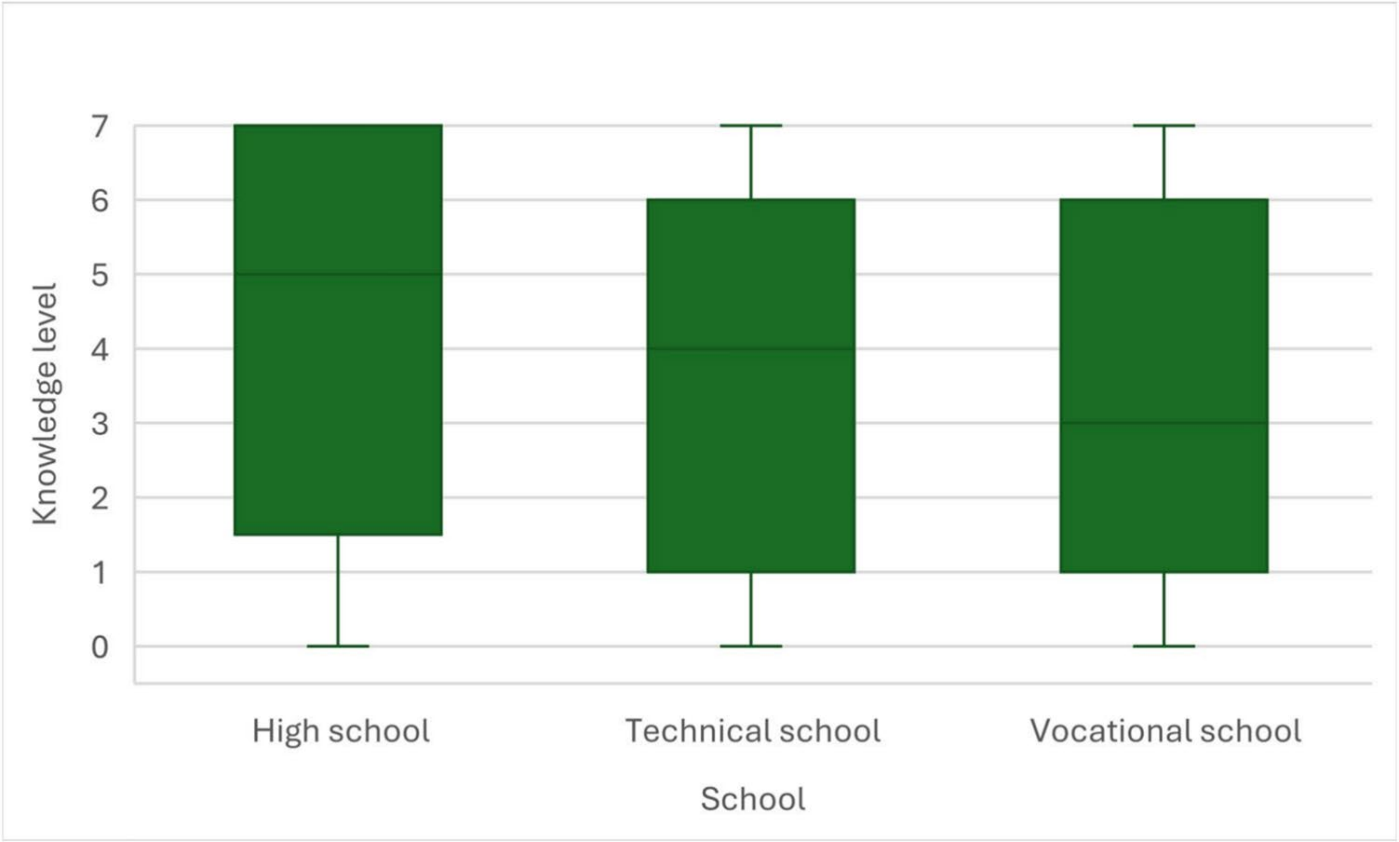
Knowledge level in high school, technical school and vocational school students

To verify the difference in knowledge level between high school, technical school and vocational school students, we performed Kruskal–Wallis non-parametric ANOVA test, that happened to be statistically significant, *H*(2) = 7.93; *p* = 0.019. Therefore, Dunn-Sidak *post-hoc* tests were performed. Two statistically significant differences were found. High school students got higher knowledge level than vocational school students (*p* = 0.028) and technical school students (*p* = 0.018). Those two groups did not differ statistically significantly from each other (*p* = 0.712). Results are presented in Fig. 4.

**Fig. 5:**
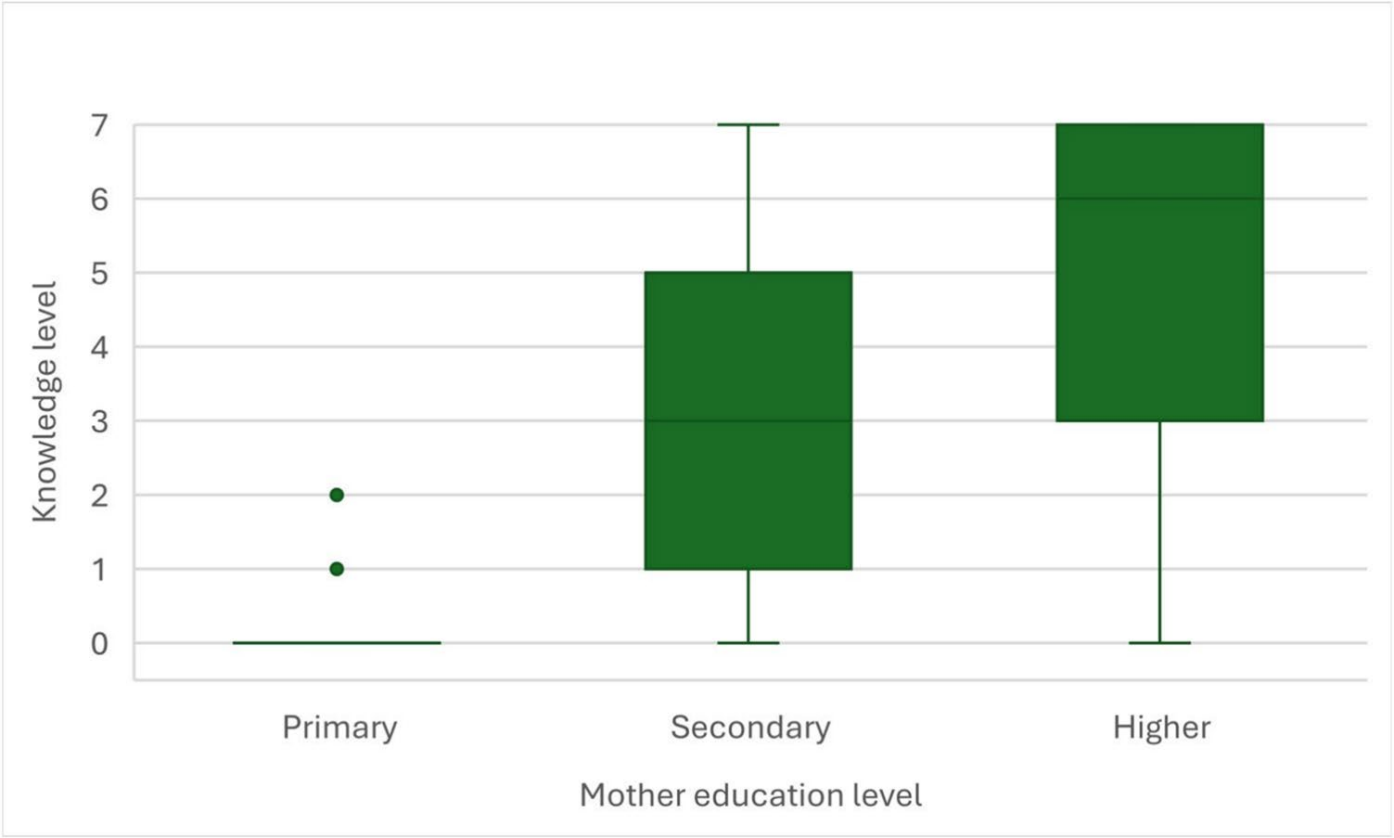
Correlation between knowledge level in secondary school students and their mother’s education level

**Fig. 6:**
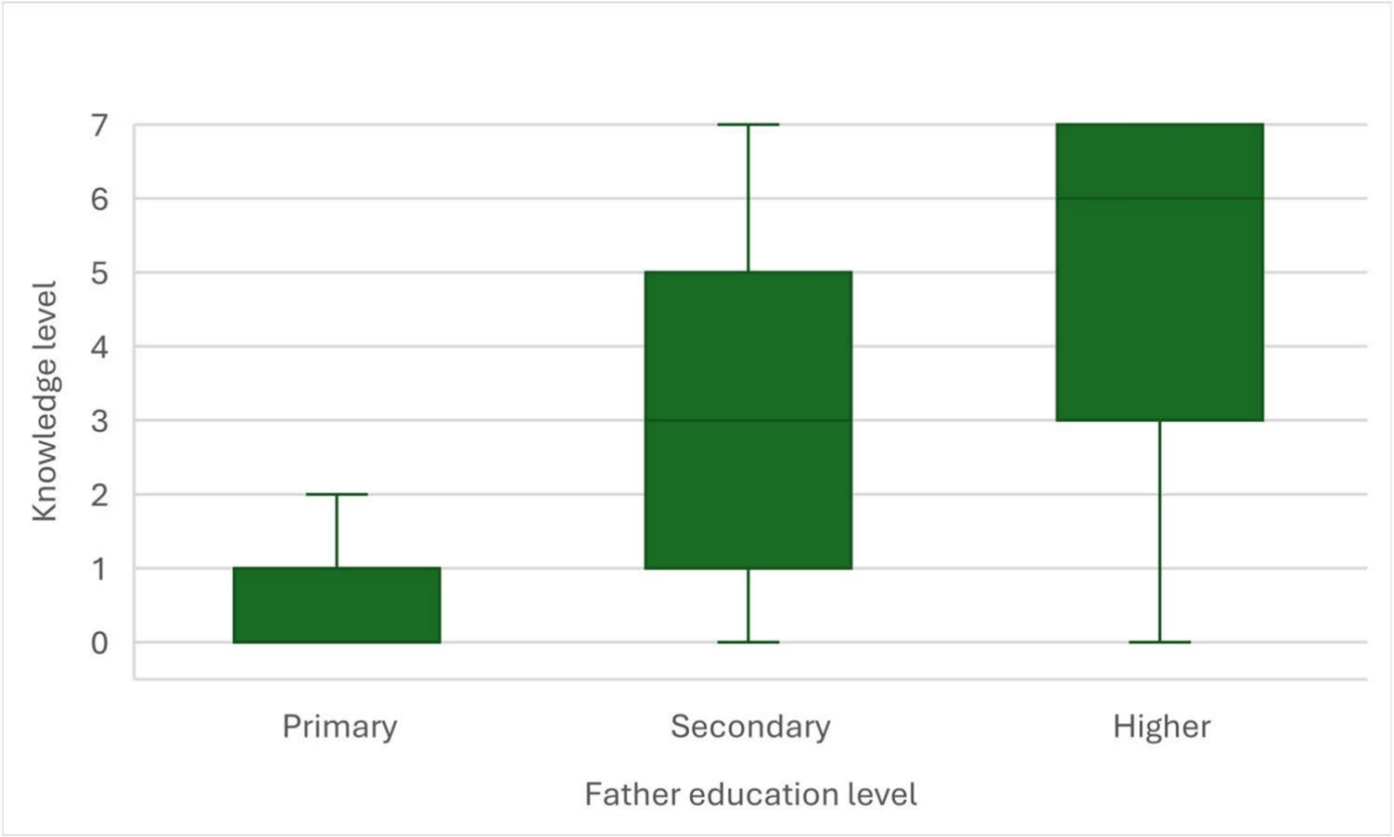
Correlation between knowledge level in secondary school students and their fathers’s education level

To verify the correlation between knowledge level of students and their parents education level, we performed Spearman’s rank correlations, that happened to be statistically significant for both level of education of mother (*r*_*s*_ = 0.48; *p* < 0.001; Fig. 5) and father (*r*_*s*_ = 0.45; *p* < 0.001; Fig. 6). The higher was the education level of participants’ parents, the higher was their own knowledge level. Both correlations were moderately strong.

**Fig. 7:**
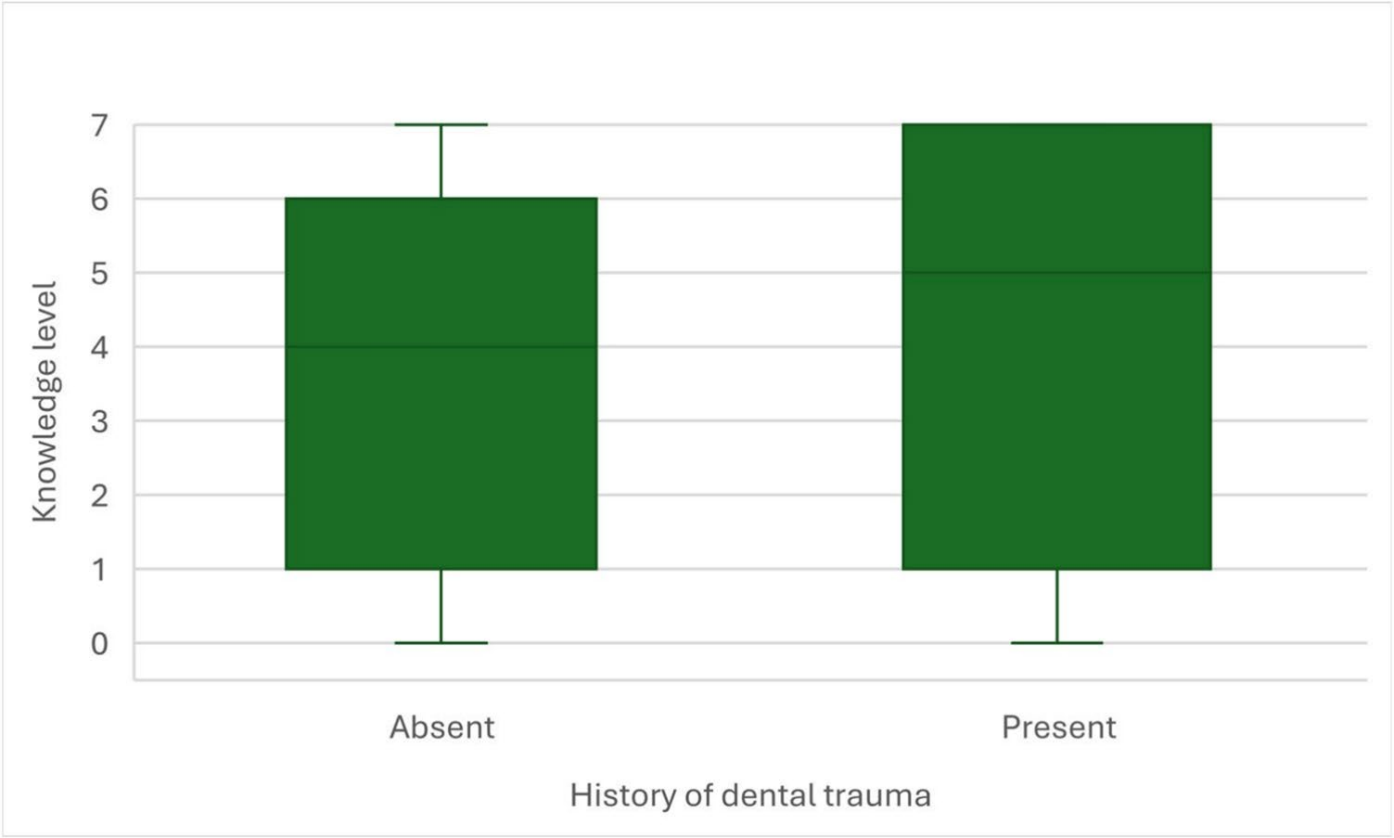
Knowledge level in secondary school students with and without history of dental trauma

In the last step, we verified the difference in knowledge level between students with and without history of dental trauma. We performed *U* Mann–Whitney’s test, that appeared to be non-statistically significant, *U* = 6704.5; *Z* = 1.64; *p* = 0.101. Therefore, we must conclude, that history of dental trauma did not differentiate the level of students’ knowledge (Fig. 7).

## Discussion

The literature indicates that nearly half of TDIs occur in school settings, making it essential that teachers, coaches, and other school staff know how to respond appropriately when such incidents happen. Adolescents aged 14–19 years should also be equipped with basic, practical guidance on what to do if dental trauma occurs, as they are often witnesses—or even first responders—before adult help arrives. In our study, 79.8% of surveyed students reported having attended a first-aid course in the past; however, only 7.1% recalled that the course included information on the management of traumatic dental injuries. This suggests that dental trauma is commonly treated as a low-priority topic within first-aid education and is therefore frequently overlooked. Given how often dental injuries affect children and adolescents, this gap in training is difficult to justify and highlights a clear need to incorporate TDI first-aid protocols into standard school-based first-aid curricula. In the literature there are many studies considering knowledge of TDI management among schoolteachers and sport coaches. In most of these researches the knowledge in this topic is insufficient. D. Tahririan et al. reported that only 10,9% of surveyed teachers had participated in a first aid course where TDI management was explained (Tahririan et al. 2022). This is a very small number considering that 61.7% of all respondents had a history of dealing with dental trauma. There was no significant difference in knowledge between teachers who experienced dental trauma in the past and those who had not. In our study, students who had suffered dental trauma in the past did not show significantly better knowledge of TDI management than those without such experience, indicating that previous trauma did not differentiate the level of students’ knowledge. This contrasts with several studies among teachers and other adult groups, where previous experience with dental trauma or prior education on TDI was identified as a positive predictor of better knowledge (Antunes et al. 2016; Fux-Noy et al. 2011; Kurnaz and Bayraktar 2021; Ivanda et al. 2021). L. Alves Antunes et al. reported that 16.6% of surveyed teachers had witnessed dental trauma, while 23.9% had received first-aid training; however, only 4.1% of those trained stated that dental trauma management was covered during the course (Antunes et al. 2016). Similarly, in a study from Israel by A. Fux–Noy et al., although 76.8% of teachers had attended a first-aid course, just 3.7% recalled any content related to dental first aid (Fux-Noy et al. 2011). In both studies, previous experience with dental trauma emerged as an important predictor of better knowledge: teachers who had either prior education on TDI or direct experience with such injuries were more likely to answer management-related questions correctly. Consistent findings were reported by S. Kurnaz et al., where 49.9% of teachers had received first-aid training, but only 2.2% had been instructed in first aid for dental emergencies, despite 28.1% having already encountered TDI at school (Kurnaz and Bayraktar 2021). Likewise, S. Ivanda et al. demonstrated that prior experience with dental trauma significantly improved participants’ knowledge compared with those without such experience (Ivanda et al. 2021). 76,7% of all responders in this study stated that it is necessary to seek medical consultation after dental trauma. Similar results were obtained in Hong Kong, where 73.1% of teachers correctly stated that a dental trauma patient should go for treatment immediately (Young et al. 2012). 65% of Singapore teachers and 44,8% of surveyed teachers in Saudi Arabia have also shared the same opinion (Al-Obaida 2010; Sae-Lim and Lim 2001).

L. Andersson et al. conducted a study on 221 Kuwaiti schoolchildren and found that 30.3% had experienced dental trauma in the past (Andersson et al. 2006). Numerous studies have also shown that dental injuries are more common in boys than in girls (Parvini et al. 2023; Paul and Acharya 2022; Lima et al. 2024). In our study, 64.3% of all respondents reported a history of dental trauma; among these, 61% were male and 39% were female, confirming the male predilection for TDI and aligning our results with previous epidemiological data. The higher risk among boys is often attributed to greater engagement in vigorous physical activities and contact sports, as well as more risk-taking behavior. The discrepancy between the overall prevalence observed in our sample and that reported by Andersson et al. may additionally be explained by the difference in age groups: while their study included children aged 7–12 years, our sample consisted of adolescents aged 14–19 years. This may suggest that a substantial proportion of traumatic dental injuries occur after the age of 12, when young people are more likely to participate in organized sports and lead a more active lifestyle.Another factor associated with knowledge level in our study was the type of school. Students attending general secondary (high) schools achieved significantly better TDI knowledge scores than those from technical or vocational schools. This difference may reflect the more academic orientation of high schools, where the curriculum often includes extended courses in biology and health education and where students may be more accustomed to theoretical learning and exam-type questions. It is also possible that high-school students, on average, have higher health literacy and are more receptive to health-related information than their peers in vocational tracks. Although we did not collect detailed data on school curricula or individual educational aspirations, these findings suggest that future educational programmes on TDI should be tailored to reach students from all school types and may need to be specially adapted to the needs of technical and vocational schools. To our knowledge, no previous studies have specifically compared TDI-related knowledge among students from different types of secondary schools (general, technical and vocational). Most published research has focused on other institutional characteristics, such as public versus private or urban versus rural schools, usually among teachers rather than students, and has generally reported similarly low levels of knowledge across school types with only minor differences in specific items (Paul and Acharya 2022).

Participation in sports is widely recognised as a major risk factor for TDI. The prevalence of dental trauma has been estimated at 11.38% in contact sports and 5.24% in non-contact sports (Lima et al. 2024). In our sample, 63.9% of respondents reported practising sports outside school physical education classes; among them, 20.1% played football, 17.9% volleyball, 13.4% basketball, 2.8% handball and 15.2% martial arts. Several studies have shown that the knowledge of emergency management of dental injuries among sports coaches remains insufficient (Vliet et al. 2022). Coaches with paramedical training demonstrate significantly better awareness compared with those without such a background, yet only 53% reported knowing how to manage an injury to a permanent tooth. This underlines the need for targeted education of coaches in dental first aid. Another crucial preventive measure is the use of mouthguards during contact sports. Various types are available, including stock, boil-and-bite and custom-made devices. A study by D. Gawlak et al. demonstrated that custom mouthguards, particularly those fabricated using injection moulding, offer superior comfort and protective properties compared with standard types (Gawlak et al. 2015). Moreover, consistent use of a mouthguard has been reported to reduce the risk of complications following dental trauma (Ilia et al. 2014; Hashim 2011).In numerous studies, teachers have expressed dissatisfaction with their level of knowledge regarding TDI management (Al-Jundi et al. 2005; Al-Asfour et al. 2008). Adel Al-Asfour et al. evaluated teachers’ knowledge twice—before and after a brief lecture on dental first aid—and demonstrated that even a short educational session can significantly improve understanding and constitutes an effective, efficient method of raising awareness of TDI emergency procedures (Lieger et al. 2009). Other low-cost educational strategies have also shown promise. O. Lieger et al. distributed educational posters on TDI first aid to 100 Swiss schools and found that teachers working in areas where posters were displayed had better knowledge than those in regions without such a campaign (Razeghi et al. 2020). Similar approaches have been tested in Iran, where an interventional study among mothers of 8-year-old schoolchildren compared education via poster, pamphlet and a control group; both posters and pamphlets were equally effective in improving knowledge of TDI management (Al-Asfour and Andersson 2008). Likewise, a simple leaflet containing essential information has been shown to be a useful tool for conveying key messages and enhancing laypeople’s understanding of how to deal with dental injuries (Abulhamael et al. 2025). In our survey, female students achieved significantly higher scores in the TDI knowledge test than male students (Fig. 2). Similar gender-related differences have been described in some recent papers. Abulhamael et al. (Abulhamael et al. 2025) reported that female medical, nursing and pharmacy students in Saudi Arabia were about three times more likely than males to have adequate knowledge of dental trauma and the ToothSOS mobile application. Bhusari et al. (Bhusari et al. 2023) also observed that female school teachers had a more positive attitude towards emergency management of dental trauma than their male colleagues, although gender differences in knowledge were not statistically significant. This results therefore add to a body of evidence suggesting that, at least in some adolescent and young adult populations, females may be more engaged with health-related topics and more receptive to educational messages about dental emergencies.

The present findings have several practical implications. First, the generally low level of knowledge regarding first-aid management of traumatic dental injuries among secondary school students indicates that dental trauma should be explicitly included in school-based first-aid workshops. Short, structured educational interventions presented by dentists could be implemented during health education or physical education classes. Given the high proportion of respondents engaged in sports, collaboration with sports clubs and coaches may be particularly effective for promoting the use of mouthguards and improving awareness of appropriate emergency procedures after dental trauma. Additionally, simple tools such as posters, leaflets or digital materials summarizing key steps in TDI management could be distributed in schools to reinforce learning and provide easily accessible guidance in case of an emergency.

## Conclusions

Secondary school students from central Poland showed limited knowledge of first-aid management of traumatic dental injuries, despite many of them having previously attended general first-aid courses. Moreover, previous personal experience of traumatic dental injury was not associated with better knowledge of TDI management, indicating that such experiences alone do not improve first-aid competence and that structured education is necessary. These results highlight the need to incorporate dental trauma into standard first-aid training in schools and to develop targeted educational strategies for adolescents and those supervising them, including teachers and sports coaches. Implementing low-cost educational measures may improve emergency management of TDI and ultimately contribute to better long-term outcomes for affected teeth.

## Data Availability

The datasets generated and/or analyzed during the current study are available from the corresponding author on reasonable request.
